# Development and validation of a CT-based radiomics nomogram for predicting overall survival in primary tracheal malignancy

**DOI:** 10.3389/fonc.2026.1609920

**Published:** 2026-04-15

**Authors:** Shuchao Wang, Yu Zhang, Zhongfeng Xie, Limin Xue, Jinwei Qiang

**Affiliations:** 1 Department of Radiology, Shanghai Tenth People′s Hospital, Tongji University, Shanghai, China; 2 Department of Radiology, Shanghai Chest Hospital, Shanghai Jiaotong University, Shanghai, China; 3 Department of Radiology, First Affiliated Hospital, School of Medicine, Zhejiang University, Hangzhou, China; 4 Department of Radiology, Jinshan Hospital, Fudan University, Shanghai, China

**Keywords:** computed tomography, nomogram, overall survival, radiomics, tracheal malignancy

## Abstract

**Objectives:**

Primary tracheal malignancies (PTMs) are rare and histologically diverse, leading to complex and contentious prognostic factors. Consequently, accurately predicting the survival outcomes of these patients is challenging. This study aimed to develop a radiomics-based prognostic model for individual prediction of survival risk in PTM patients.

**Methods:**

A total of 115 patients with PTM were reviewed retrospectively and divided into the training cohort (n = 85) and validation cohort (n = 30). Radiomics features associated with overall survival (OS) were selected using the least absolute shrinkage and selection operator (LASSO) method and combined to form the radiomics score (Radscore). Multivariable analyses were used to identify clinical and CT features as independent risk factors for OS. Radscore and identified risk factors were combined to construct a radiomics nomogram. The predictive efficacy and clinical net benefit of the prognostic models were assessed using the C-index and decision curve analysis (DCA).

**Results:**

Seven radiomics features were selected by LASSO to form a Radscore. Longitudinal length was identified as an independent prognostic factor for OS. Compared with longitudinal length (C-index: 0.59; 95% confidence interval [CI]: 0.47-0.70), both the Radscore (C-index: 0.75; 95% CI, 0.63-0.87) and nomogram (C-index: 0.79; 95% CI, 0.69-0.88) demonstrated better predictive performance and were confirmed in the validation cohort. In addition, DCA indicated that both the Radscore and nomogram provided favorable clinical net benefits.

**Conclusions:**

The CT-based nomogram, which combined Radscore and longitudinal length, could individually predict the survival outcomes of PTM patients, aiding clinical decision-making.

## Introduction

Primary tracheal malignancy (PTM), a rare condition, which accounts for about 1-2% of all tumors and 0.5% of malignant tumors of the upper respiratory tract, with an incidence of about 1 new case per 1,000,000 people per year (1–3). PTM typically occurs between the cricoid cartilage and the carina of the trachea, with a tendency to develop at the junction of the tracheal cartilage ring and membrane (4). The most common histological subtypes are squamous cell carcinoma (SCC) and adenoid cystic carcinoma (ACC). Reports suggest that SCC is more prevalent among men and smokers, whereas ACC shows no correlation with gender or smoking (5–7). Early and accurate diagnosis of PTM is challenging due to its insidious onset and non-specific manifestation (8, 9). The 5-year overall survival (OS) of patients with PTM ranges from 15% to 45% (10, 11). Surgery is considered the best treatment modality for PTM, but achieving complete resection with negative margins is often challenging (12). Local recurrence and distant metastasis are major contributors to treatment failure (10, 13). Several retrospective studies suggest that patients with PTM may benefit from adjuvant radiotherapy and chemotherapy, but their efficacy remains controversial (3, 14). In addition, due to its low incidence, no widely accepted staging system has been developed to comprehensively predict the prognosis of PTM.

Computed tomography (CT) allows assessment of the extent of local infiltration, invasion of surrounding tissues and lymphatic and distant metastases, making it the mainstay of PTM diagnosis and staging in conjunction with tracheoscopy (11, 14, 15). As a promising quantitative analysis technique, CT-based radiomics models have been employed to diagnose a wide range of tumors, monitor treatment response, and predict survival outcomes (15).

To date, the application of a radiomics approach to explore the prognostic value of CT for PTM has not been reported. Therefore, the aim of this retrospective study was to develop a radiomics-based prognostic model by integrating clinical data and CT images of patients with PTM to evaluate the survival risk of patients.

## Methods

### Study population

Approval from the ethical review board of our institution and a waiver for informed consent were obtained for this retrospective study. From January 2012 to December 2021, clinical-pathologic data and CT images of patients with PTM were collected and reviewed retrospectively at our institution. We excluded patients who met the following criteria: (a) patients lacking complete clinical-pathologic data; (b) patients with a history of other malignancies; (c) patients who underwent CT scanning more than 1 month before surgery; (d) patients with benign, recurrent, or metastatic tracheal tumors; (e) patients who received preoperative tracheoscopic intervention, radiotherapy, or chemotherapy, and (f) patients with lesions that could not be accurately segmented. Finally, a total of 115 patients with PTM were enrolled in this study and randomly assigned to the training and validation cohorts in a 7:3 ratio. Overall survival (OS), used as an indicator of survival outcomes, was defined as the time interval from the date of first treatment to death from any cause or to the last follow-up visit.

### CT scanning and image acquisition

Chest or neck CT scans of 115 patients with PTM were performed using multidetector-row CT scanners (Brilliance, Ingenuity, iCT, Philips, Cleveland, USA) at our institution. The scanning parameters were as follows: 120 kV, 250 mA, collimation of 0.625 mm, pitch of 0.984, field of view of 400×400 mm, a reconstructed slice thickness of 1mm, matrix of 512×512, standard reconstruction algorithm, and filter function C. Image analysis was performed independently by two junior radiologists (S.C.W. and L.M.X, with 6 and 3 years of experience in chest imaging diagnosis, respectively), and any discrepancies were resolved by consensus. Pathologic and clinical information of PTM patients was processed in a blinded manner. CT features evaluated included: longitudinal length, transverse length, location, morphology, growth pattern, contour, border, and homogeneity of the lesion.

### Image segmentation and feature extraction

Radiomics features were extracted from CT images without any preprocessing or normalization. The regions of interest (ROIs) were outlined slice by slice along the border of the lesion on axial images using 3D-Slicer software (https://www.slicer.org) to generate a three-dimensional volume of interest (VOI). Subsequently, the radiomics features of the VOI were automatically extracted using Pyradiomics (https://pypi.org/project/pyradiomics/), an in-house software package developed in Python. Finally, a total of 944 radiomics features were extracted from each lesion, including 2D and 3D shape features, first-order features, texture features, as well as Laplacian of Gaussian (LoG) and wavelet-based features. The texture features comprised gray level co-occurrence matrix (GLCM), gray-level size zone matrix (GLSZM), gray-level run-length matrix (GLRLM), and gray-level dependence matrix (GLDM). Additionally, redundant features with a correlation coefficient greater than 0.9 were removed through Pearson correlation analysis.

### Feature selection, model construction and validation

Radiomics features associated with OS were selected using the least absolute shrinkage and selection operator (LASSO) Cox regression model and 10-fold cross-validation. Then, the selected features and their corresponding weighting coefficients were linearly combined to obtain the Radiomics Score (Radscore) as a radiomics feature signature.

The optimal cutoff value for Radscore associated with OS in the training cohort was calculated using X-tile software (version 3.6.1, Yale University School of Medicine, New Haven, CT, USA) and validated in the validation cohort. Accordingly, patients were divided into low-risk and high-risk groups, and the survival outcomes were evaluated and compared using the Kaplan-Meier survival analysis and log-rank test.

Univariable and multivariable Cox regression analyses were used to assess the association of clinical and CT characteristics with OS as independent prognostic factors in patients with PTM. A nomogram was constructed by integrating Radscore and independent prognostic factors identified by Cox regression models for individualized and quantitative assessment of survival risk in patients with PTM.

Subsequently, the predictive efficacy of Radscore, independent prognostic factors and the nomogram were evaluated and compared using the C-index and likelihood ratio test, respectively. In addition, calibration curves were plotted to assess the agreement between predicted and actual observations of the nomogram. Decision curve analysis (DCA) was used to calculate the net clinical benefit obtained from the three predictive models at different threshold probabilities. An overview of the workflow for this study is shown in **Supplementary Figure 1**.

### Statistical analysis

Continuous variables were compared using the independent samples t-test or Mann-Whitney U test, and categorical variables were compared using the chi-square test or Fisher’s exact test. LASSO logistic regression, nomogram, and DCA were generated using the “glmnet” package, “rms” and “survival” packages, and the DCA package (https://github.com/ddsjoberg/dca), respectively. All the statistical analyses were conducted using SPSS software (https://www.ibm.com/spss/statistics, Version 23.0.0, IBM), R language (https://www.r’-project.org, Version 4.0.4), and RStudio (https://rstudio.com). A two-sided *p-* value < 0.05 indicated a statistically significant difference. Bootstrap validation with 1000 resamples was performed to evaluate potential model overfitting and assess the stability of the Radscore cutoff.

## Results

### Clinical and CT features

A total of 115 patients with PTM (median age 50 years; interquartile range [IQR], 24-85 years; 47 women) were enrolled for radiomics training and internal validation. In addition to the predominant adenoid cystic carcinoma (ACC) (n = 77) and squamous cell carcinoma (SCC) (n = 27), other histologic types included mucoepidermoid carcinoma (n = 3), small cell carcinoma (n = 3), epithelial-myoepithelial carcinoma (n = 2), Hodgkin’s lymphoma (n = 1), MALT lymphoma (n = 1), and inflammatory myofibroblastic tumor (n = 1). Treatment modalities included radical surgery alone in 44 patients (38.3%), radical surgery followed by adjuvant radiotherapy or chemotherapy in 46 patients (40.0%), and non-surgical treatments (radiotherapy and/or chemotherapy and/or tracheoscopic intervention) in 25 patients (21.7%). The median follow-up time for all PTM patients was 51 months (IQR, 3-129 months), with 31 cases had an endpoint event (death), and the median OS duration was 93 months, with 1-year, 3-year, and 5-year survival rates of 97.4%, 80.8%, and 61.9%, respectively. In addition, in the training (n = 85) and validation (n = 30) cohorts, the median age of patients was 48 years and 53 years, respectively, and the median OS duration was 93 months and 65 months (*p* = 0.470). As detailed in **Table 1**, except for the histologic type (*p* = 0.021), other clinical and CT characteristics were not significantly different (*p* > 0.05) between the training and validation cohorts.

**Table 1:** Clinical and CT characteristics of all PTM patients, training and validation cohorts.

Variables	Total	Training cohort	Validation cohort	*p-*value
	(n=115)	(n=85)	(n=30)	
Age (years)	50.0 (24-85)	48.0 (26-78)	53.0 (24-85)	0.304
Gender				0.329
Male	68 (59.1%)	48 (56.5%)	20 (66.7%)	
Female	47 (40.9%)	37 (43.5%)	10 (33.3%)	
Histological subtype				**0.021** ^a^
Adenoid cystic carcinoma	77 (67.0%)	63 (74.1%)	14 (46.7%)	
Squamous cell carcinoma	27 (23.5%)	16 (18.8%)	11 (36.7%)	
Others	11 (9.6%)	6 (7.1%)	5 (16.7%)	
Treatment				0.133
Surgery	44 (38.3%)	29 (34.1%)	15 (50.0%)	
Surgery with adjuvant therapy	46 (40.0%)	34 (40.0%)	12 (40.0%)	
Non-surgical treatment	25 (21.7%)	22 (25.9%)	3 (10.0%)	
Longitudinal length (mm)	33.0 (7-86)	31.0 (10-86)	33.5 (6-49)	0.669
Transverse length (mm)	18.0 (5-44)	17.0 (5-44)	19.0 (6-43)	0.180
Location				0.401
Proximal trachea	26 (22.6%)	20 (23.5%)	6 (20.0%)	
Middle trachea	42 (36.5%)	28 (32.9%)	14 (46.7%)	
Distal trachea	47 (40.9%)	37 (43.5%)	10 (33.3%)	
Shape				1.000
Broad base nodule	63 (54.8%)	46 (54.1%)	17 (56.7%)	
Narrow base nodule	6 (5.2%)	5 (5.9%)	1 (3.3%)	
Annular thickened wall	46 (40%)	34 (40.0%)	12 (40.0%)	
Growth mode				0.051
Intraluminal	52 (45.2%)	43 (50.6%)	9 (30.0%)	
Extraluminal/Transmural	63 (54.8%)	42 (49.4%)	21 (70.0%)	
Contour				0.286
Smooth	67 (58.3%)	52 (61.2%)	15 (50.0%)	
Lobular	48 (41.7%)	33 (38.8%)	15 (50.0%)	
Border				0.731
Well defined	87 (75.7%)	65 (76.5%)	22 (73.3%)	
Poorly defined	28 (24.3%)	20 (23.5%)	8 (26.7%)	
Homogeneous				0.129
Yes	67 (58.3%)	46 (54.1%)	21 (70.0%)	
No	48 (41.7%)	39 (45.9%)	9 (30.0%)	
Median overall survival (months)	93.0	93.0	65.0	0.470

^a^ Statistically significant at *p* < 0.05.Bold values indicate statistical significance (p < 0.05).

### Radiomics feature selection and signature construction

**Figure 1:**
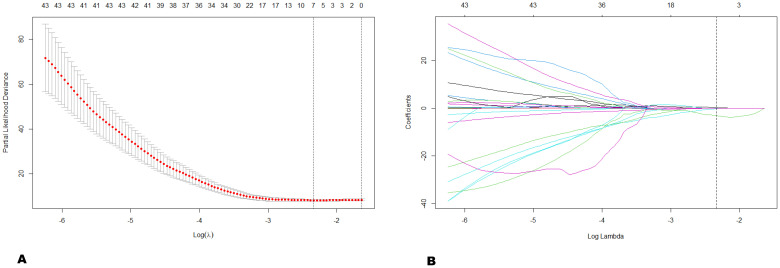
LASSO Cox regression selecting radiomics features. **(A)** Tuning parameter λ of the LASSO model was selected using the minimum partial likelihood deviation (PLD) as a criterion for 10-fold cross-validation. The vertical dashed line on the left was λ (λ. min) at the minimum of the PLD, and the vertical dashed line on the right was λ (λ. 1se) with the PLD within 1 standard error. Finally, 0.097 and -2.334 were chosen as the optimal value for λ. min and Log (λ), respectively. **(B)** Distribution of LASSO coefficients for 318 features. The vertical dashed line implied that LASSO selected seven features with non-zero coefficients when λ was the optimal value.

**Figure 2:**
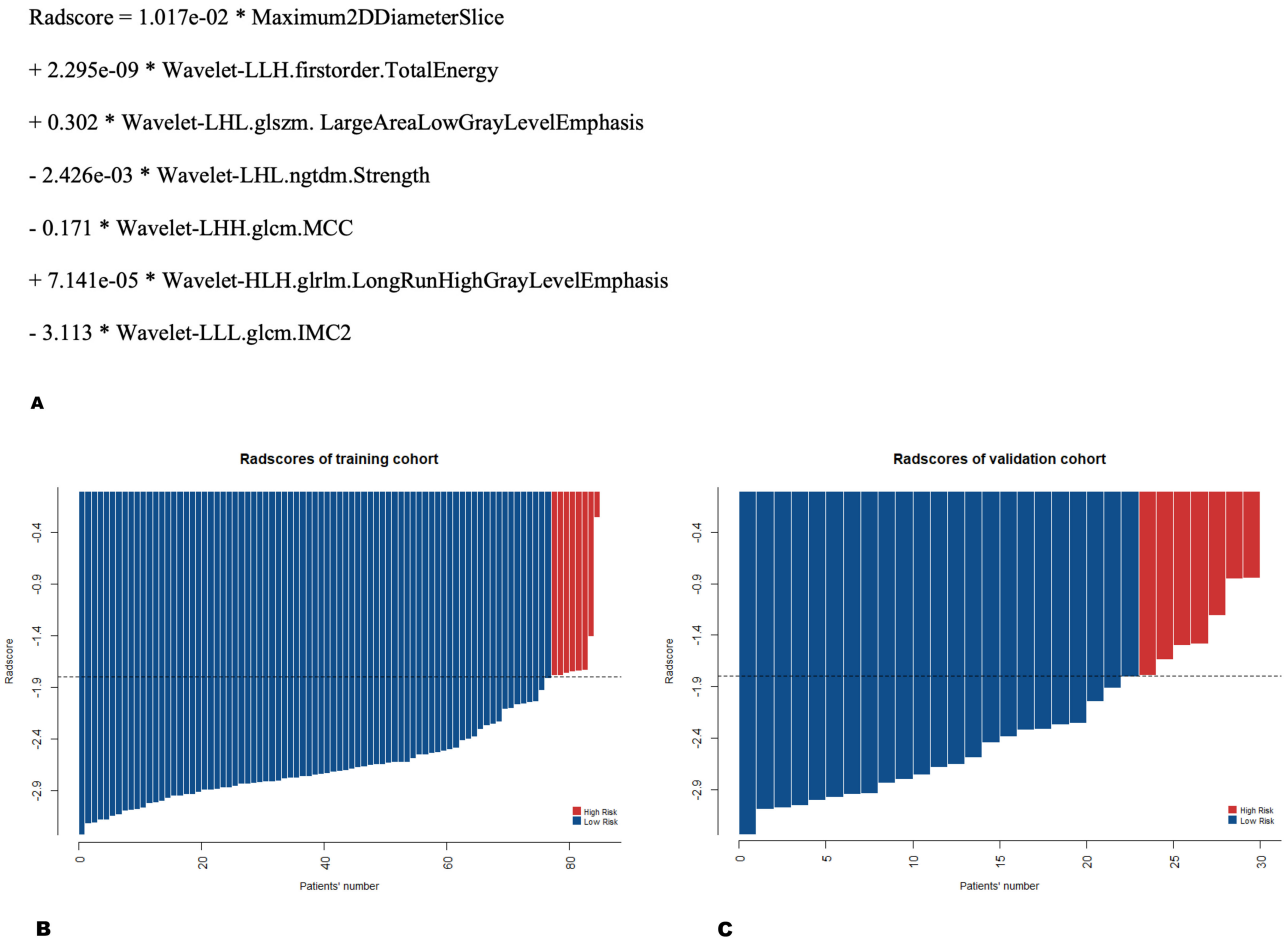
Equation **(A)** and distribution **(B, C)** of Radscore for each patient. Radscore = -1.80 was used as the cutoff value for 5-year overall survival to divide patients into high- and low-risk groups, with red and blue representing the Radscore of patients in the high- and low-risk groups, respectively. **(B)** The training cohort. **(C)** The validation cohort.

After tumor segmentation of the included patients, 944 features were extracted from the outlined ROIs. To ensure stability and reproducibility of the radiomics features, 626 redundant features (intraclass correlation coefficient > 0.9) were removed using Pearson correlation analysis. Subsequently, seven features with non-zero coefficients were selected by the LASSO regression model from the remaining 318 features. These selected features included one 2D shape feature and six wavelet transform-based features (one first-order feature, one GLSZM feature, one NGTDM feature, two GLCM features, and one GLRLM feature) (**Figure 1**). These features and their respective weighting coefficients were linearly combined to obtain the Radscore, the equation for which used to predict survival risk for each patient, as shown in **Figure 2A**.

**Figure 3:**
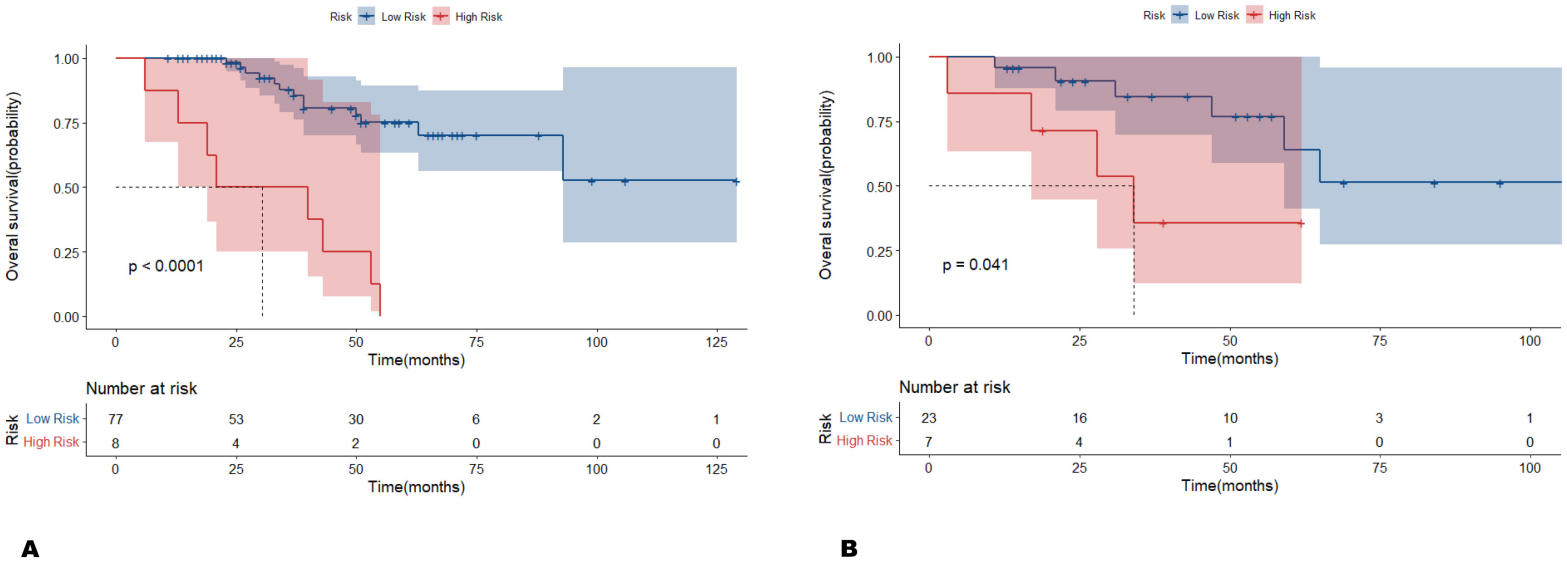
Kaplan-Meier survival curves, with red and blue representing the high- and low-risk groups, respectively. The log-rank test showed a significant difference in 5-year overall survival between patients in the high- and low-risk groups within both the **(A)** training cohort and **(B)** validation cohort (p < 0.001, p = 0.041).

According to the optimal cutoff value of -1.80 for Radscore in the training cohort, determined by X-tile, patients were stratified into a high-risk group (Radscore ≥ -1.80) and a low-risk group (Radscore < -1.80). The distribution of radiomics features showed that patients with a low Radscore were generally associated with favorable OS, whereas those with a high Radscore had an increasing risk of death in both the training and validation cohorts (**Figures 2B, C**). The log-rank test indicated a statistically significant difference in 5-year OS between the high-risk and low-risk groups in both the training and validation cohorts (*p* < 0.001 and *p* = 0.041, respectively) (**Figure 3**). The C-index of Radscore was 0.751 (95% CI: 0.634, 0.867), indicating moderate accuracy.

### Nomogram construction and evaluation

Univariable analysis indicated that the survival outcomes of patients with PTM were substantially associated with clinical and CT characteristics, including age (*p* = 0.038), transverse length (*p* = 0.006), and growth pattern (*p* = 0.007) (**Table 2**). Interestingly, the selection of all factors with *p* < 0.1 for multivariable Cox regression analysis suggested that longitudinal length was the only independent predictor of OS (hazard ratio = 1.034; 95% CI: 1.002, 10.066; *p* = 0.037).

**Table 2:** Univariable and multivariable Cox regression analyses for clinical and CT characteristics.

Variables	Univariable			Multivariable		
	HR	95% CI	*p*-value	HR	95% CI	*p*-value
Age	1.039	1.002-1.077	0.038^a^	1.038	0.988-1.091	0.143
Gender	0.461	0.179-1.191	0.110			
Histological subtype			0.060			0.132
Adenoid cystic carcinoma						
Squamous cell carcinoma	3.435	1.342-8.797	0.010	3.475	1.038-11.632	0.043
Others	1.626	0.209-12.657	0.643	2.602	0.245-27.670	0.428
Treatment			0.100			
Surgery						
Surgery with adjuvant therapy	1.151	0.386-3.430	0.801			
Non-surgical treatment	2.972	1.012-8.726	0.048			
Longitudinal length	1.020	0.998-1.043	0.073	1.034	1.002-1.066	**0.037** ^a^
Transverse length	1.064	1.018-1.113	**0.006** ^a^	1.041	0.976-1.111	0.221
Location			0.700			
Proximal trachea						
Middle trachea	1.201	0.351-4.113	0.771			
Distal trachea	1.597	0.500-5.103	0.430			
Shape			0.800			
Broad base nodule						
Narrow base nodule	2.033	0.248-16.666	0.509			
Annular thickened wall	1.019	0.409-2.538	0.967			
Growth mode	4.025	1.472-11.010	0.007^a^	1.492	0.366-6.087	0.577
Intraluminal						
Extraluminal/Transmural	4.013	1.467-10.975	0.007			
Contour	1.273	0.519-3.124	0.598			
Border	0.964	0.339-2.739	0.945			
Homogeneity	0.866	0.364-2.060	0.745			

^a^ Statistically significant at *p* < 0.05. HR: hazard ratio; CI: confidence interval.Bold values indicate statistical significance (p < 0.05).

**Figure 4:**
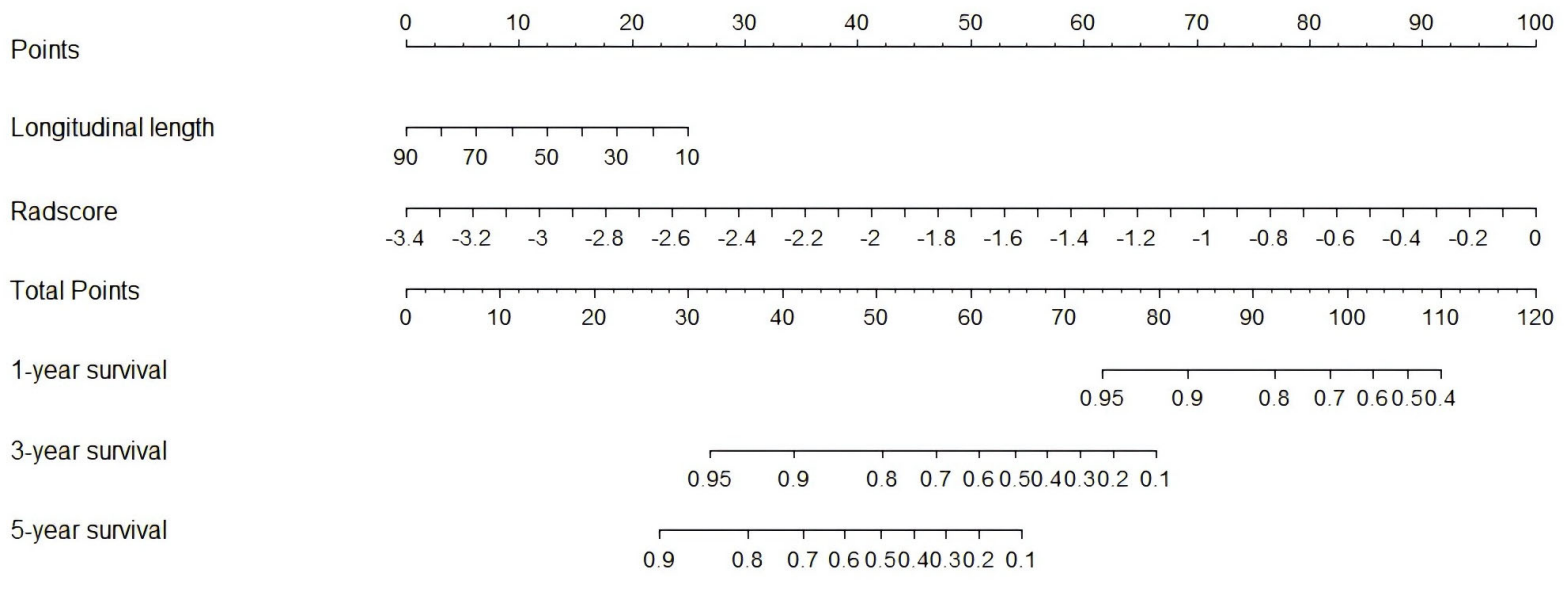
Construction of radiomics nomogram combining independent prognostic feature (longitudinal length) and Radscore in the training cohort.

**Figure 5:**
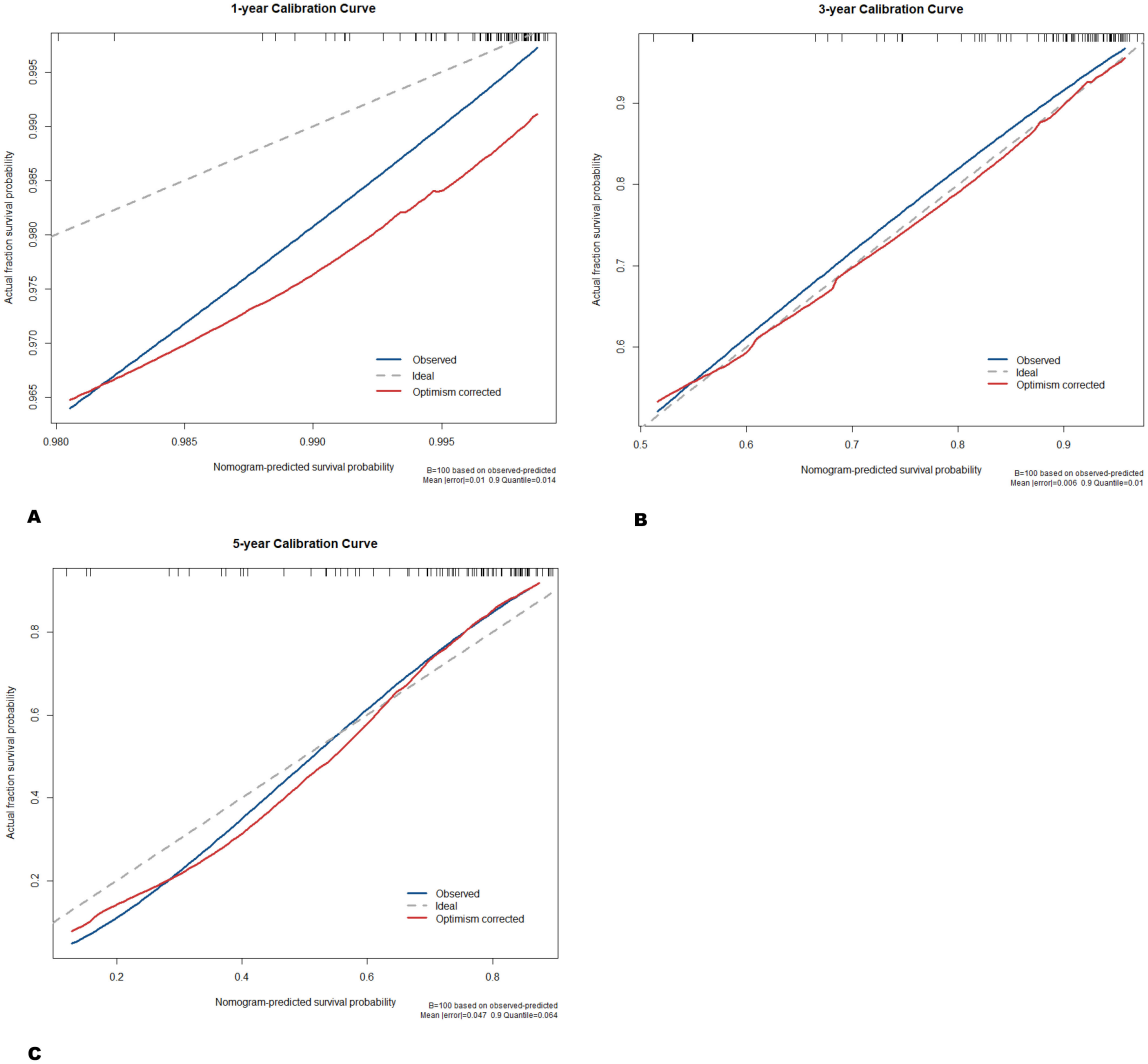
Calibration curves of radiomics nomogram in the training cohort. **(A)** 1-year overall survival. **(B)** 3-year overall survival. **(C)** 5-year overall survival.

**Figure 6:**
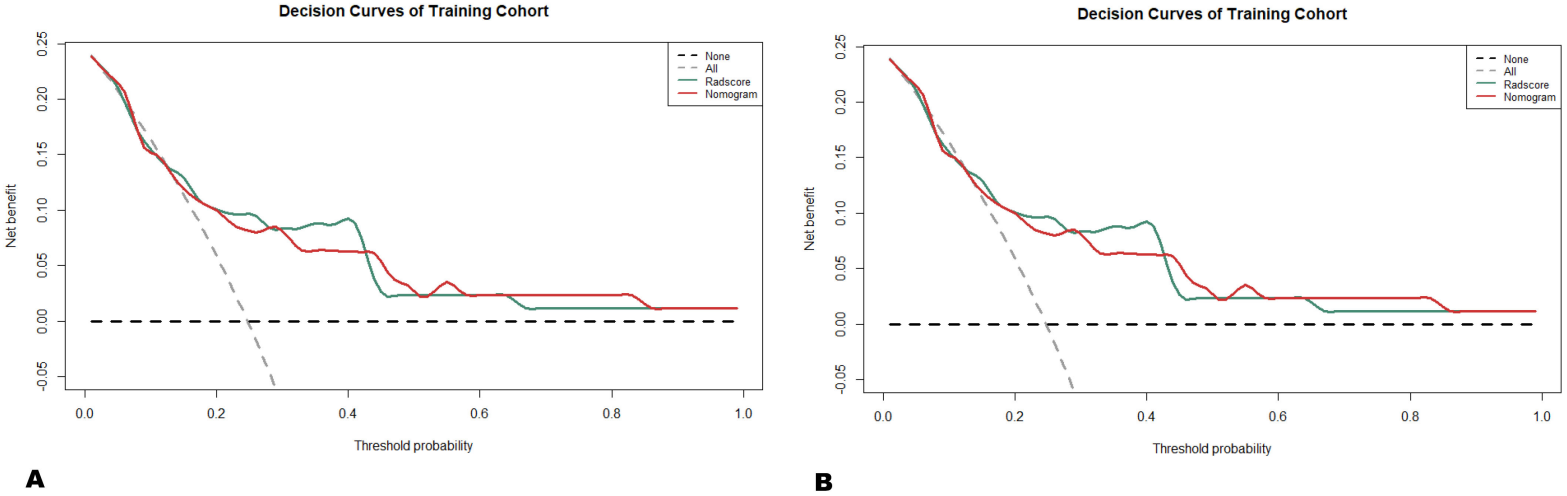
Decision curves for predicting 3- and 5-year overall survival (OS) for nomogram and Radscore. The black dashed line assumed that no patients died, and the gray dashed line assumed that death occurred in all patients. When the threshold probabilities of the **(A)** training and **(B)** validation cohorts were within 0.10-0.85 and 0.14-0.42, respectively, nomogram for the prediction of OS had net clinical benefits.

Subsequently, a nomogram was generated by combining the radiomics signature (Radscore) and clinical-imaging model (longitudinal length) to predict the 1-year, 3-year, and 5-year survival risks in patients with PTM (**Figure 4**). The calibration curves showed that the predicted 1-year, 3-year, and 5-year OS probabilities of the combined model had favorable concordance with the actual observations (**Figure 5**). Compared with the longitudinal length alone (C-index: 0.59; 95% CI, 0.47-0.70), both Radscore (C-index: 0.75; 95% CI, 0.63-0.87) and the combined model (C-index: 0.79; 95% CI, 0.69-0.88) exhibited better predictive performance for survival risks in patients with PTM (both *p* < 0.001) (**Table 3**). Similar results were observed in the validation cohort, confirming the additional prognostic performance of Radscore (**Table 3**). Considering the potential influence of histological heterogeneity, subgroup analysis was performed in patients with ACC. Kaplan-Meier survival analysis showed that patients with a high Radscore had significantly worse overall survival than those with a low Radscore (log-rank *p* = 0.018, **Supplementary Figure 2**). Additionally, we assessed the clinical usefulness of Radscore and the nomogram in predicting 3-year and 5-year OS using DCA, which indicated that both models achieved favorable net benefits (**Figure 6**).

**Table 3:** Predictive efficacy of longitudinal length, Radscore and nomogram.

Variables	Training cohort		Validation cohort	
	C-index	95% CI	C-index	95% CI
Longitudinal length	0.587	0.470-0.703	0.582	0.342-0.823
Radscore	0.751	0.634-0.867	0.632	0.462-0.802
Nomogram	0.786	0.686-0.884	0.638	0.432-0.844

CI, confidence interval.

To assess the potential overfitting of the radiomics nomogram, bootstrap validation with 1000 resamples was performed. The optimism-corrected C-index was 0.76, which was close to the original C-index (0.79), indicating minimal overfitting. The hazard ratio between the high-risk and low-risk groups remained statistically significant (HR = 2.95, 95% CI: 1.80-4.50). Detailed results are shown in **Supplementary Table 1**.

## Discussion

Given the limited research on CT imaging for PTM and its unclear prognostic value, this study explored the feasibility of CT-based prognostic models in predicting 1-year, 3-year, and 5-year survival outcomes in PTM patients. We developed a radiomics signature (Radscore) based on seven radiomics features to predict the prognosis of PTM patients with various histologic types. A cutoff value of -1.80 for Radscore enabled for the stratification of survival risk. Furthermore, multivariable survival analysis identified the longitudinal length of the tumor as the sole independent predictor of OS (p = 0.037). The radiomics nomogram, integrating Radscore and longitudinal length, achieved a C-index of 0.751 in predicting OS, highlighting its strong performance as a prognostic model for patients with PTM.

Due to the rarity and histologic heterogeneity of PTM, it is challenging to assess and predict its prognosis. Two studies based on the Surveillance, Epidemiology, and End Results (SEER) database—one with 99 cases from 1988 to 2000 and another with 287 cases from 1973 to 2013—showed that the median OS and 5-year survival rates of PTM patients were 30 months and 40.0%, and 57 months and 48.9%, respectively (8, 16). In comparison, patients with PTM in our study had better survival outcomes, with a median survival time of 93 months and a 5-year survival rate of 61.9%. We hypothesize that the better prognosis of PTM patients at our institution might be associated with the availability of more frequent CT scans for early diagnosis, more individualized treatment options, and more advanced surgical approaches in recent years. Studies have shown that the longitudinal length of tracheal tumors plays a significant role in determining patient survival outcomes (17, 18). In line with these findings, our study confirmed that CT-measured longitudinal length is an independent predictor of OS in patients with PTM.

Consistent with the classification statistics of 733 tracheal tumors reported by Mallick et al. (17), in our study, among the eight histologic types of PTM, ACC was the most common (77 cases, 67.0%), followed by SCC (27 cases, 23.5%). Moreover, their study demonstrated a difference in prognosis between different histologic types of PTM, with a median survival of 165 months for patients with ACC compared to only 14 months for those with SCC. Similarly, He and Shao et al. also demonstrated that SCC had a poorer prognosis than ACC and adenocarcinoma (16, 19). After comparing the survival outcomes of ACC and other tracheal malignancies, Regnard et al. also found that ACC had a better prognosis compared to other histologic types, with 5-year and 10-year survival rates of 73% and 57%, respectively (20). In contrast, a retrospective study of 37 tracheal tumors by Youngji et al. reported that the difference in OS between ACC and SCC was not statistically significant, with 5-year survival rates of 45.7% and 41.1%, respectively (5). Similar to the findings of Youngji et al, we also observed that OS was slightly, but not significantly, worse in SCC compared to ACC or other subtypes. In addition, subgroup analysis within the ACC cohort demonstrated that the radiomics signature remained significantly associated with overall survival, suggesting that the predictive value of Radscore was not solely driven by differences in histological subtype. However, aside from ACC and SCC, survival outcomes for the other histologic types included in our cohort could not be compared separately due to the relatively small sample size.

Surgery remains the primary treatment for patients with PTM, with radiotherapy and chemotherapy serving as postoperative adjuvant options (16). In contrast, adenocarcinoma and non-Hodgkin’s lymphoma are typically more responsive to radiotherapy (21–23). Wen and Xie et al. found that postoperative radiotherapy significantly extended OS in patients with PTM by conducting paired analyses on 405 and 258 patients with tracheal tumors, respectively, using the SEER database (13, 24). Our study showed that OS was slightly better in patients who received surgery compared to those who did not, but surgery did not significantly improve survival. Furthermore, OS was not significantly extended in PTM patients treated with postoperative radiotherapy and/or chemotherapy compared to those who underwent radical surgery alone. Two retrospective studies by Webb and Yusuf et al. involving 74 and 549 patients with PTM, respectively, also demonstrated that postoperative radiotherapy did not significantly improve survival outcomes (25, 26). In contrast, Mallick et al. reported slightly longer OS in patients with tracheal SCC treated with radiotherapy alone than in surgical patients (17). To date, retrospective studies related to treatment approaches for PTM have shown conflicting results. Further prospective studies are warranted to observe and validate the prognostic significance of various treatment options for patients with PTM.

Our univariable analysis showed that extraluminal and transmural growth were associated with a worse prognosis in patients with PTM, but after multivariable adjustment, these two growth patterns were not independent indicators of OS. Additionally, a study involving 42 patients with ACC suggested that lesion invasion outside the trachea was detrimental to disease-free survival rather than OS (12). In a study evaluating PET/CT in 37 patients with PTM, Shao et al. indicated that metabolic tumor volume (MTV) > 5.19, total lesion glycolysis (TLG) > 16.94 and extraluminal invasion were independent risk factors for OS (19). However, a limitation of their study was the relatively small sample size of the cohort and the absence of longitudinal length measurement, an important morphological feature of PTM. Moreover, considering the financial burden of patients, PET/CT is not a routine examination for PTM due to its high cost. Our study demonstrated that seven CT-based radiomics features were able to predict OS in patients with PTM, with the wavelet-transformed Informational Measure of Correlation 2 (IMC 2) having the greatest weight in Radscore. IMC 2 quantifies the complexity of the texture, with a smaller value indicating a more heterogeneous texture. Therefore, we propose that the heterogeneity of texture is a risk factor for OS in PTM patients. PTM exhibits significant histological heterogeneity and varied treatment responses, which can be addressed by incorporating CT longitudinal length and radiomics-based nomogram. This prognostic model enables early and reliable assessment of patients’ survival outcomes, thus facilitating individualized treatment regimens. The performance of the nomogram was significantly better than that of longitudinal length alone but close to that of Radscore, suggesting that the radiomics features played a dominant role in predicting survival risk.

Our study has several limitations. First, the retrospective single-center design may introduce selection bias. Second, the sample size was relatively small due to the rarity of PTM, and larger multicenter cohorts are needed for external validation of the proposed model. Third, the distribution of histological subtypes was imbalanced and the sample size of some subtypes was limited, which may affect the generalizability of the findings. Finally, several clinicopathological variables were not consistently available because of the retrospective design and heterogeneous treatment strategies and were therefore not included in our study.

In conclusion, a non-invasive and effective predictive model combining CT longitudinal length and Radscore was developed to assess the survival risk of PTM patients, which may facilitate clinical decision-making and individualized treatments.

## Data Availability

The raw data supporting the conclusions of this article will be made available by the authors, without undue reservation.

## Supplementary material

Supplementary Table 1 Bootstrap validation results of the radiomics nomogram.

Supplementary Figure 1 Over-view workflow of this study.

Supplementary Figure 2 Kaplan–Meier survival curves, with red and blue representing the high- and low-risk groups, respectively. The log-rank test showed a significant difference in overall survival between patients in the high- and low-risk groups within the adenoid cystic carcinoma (ACC) cohort (p = 0.018).

## References

[1] Meyers BF Mathisen DJ . Management of tracheal neoplasms. Oncologist. (1997) 2:245–53. doi: 10.1634/theoncologist.2-4-245. PMID: 10388056

[2] Azar T Abdul-Karim FW Tucker HM . Adenoid cystic carcinoma of the trachea. Laryngoscope. (1998) 108:1297–300. doi: 10.1097/00005537-199809000-00006. PMID: 9738744

[3] Urdaneta AI Yu JB Wilson LD . Population based cancer registry analysis of primary tracheal carcinoma. Am J Clin Oncol. (2011) 34:32–7. doi: 10.1097/coc.0b013e3181cae8ab. PMID: 20087156

[4] Yang KY Chen YM Huang MH Perng RP . Revisit of primary Malignant neoplasms of the trachea: clinical characteristics and survival analysis. Jpn J Clin Oncol. (1997) 27:305–9. doi: 10.1093/jjco/27.5.305. PMID: 9390206

[5] Ahn Y Chang H Lim YS Kim HJ Kim YH Lee KS . Primary tracheal tumors: review of 37 cases. J Thorac Oncol. (2009) 4:635–8. doi: 10.1097/jto.0b013e31819d18f9. PMID: 19357541

[6] Honings J Gaissert HA Verhagen AF van Dijck JA van der Heijden HF Marres HA . Undertreatment of tracheal carcinoma: multidisciplinary audit of epidemiologic data. Ann Surg Oncol. (2009) 16:246–53. doi: 10.1245/s10434-008-0241-3. PMID: 19037701

[7] Yang H Yao F Tantai J Zhao Y Tan Q Zhao H . Resected tracheal adenoid cystic carcinoma: improvements in outcome at a single institution. Ann Thorac Surg. (2016) 101:294–300. doi: 10.1016/j.athoracsur.2015.06.073. PMID: 26431923

[8] Bhattacharyya N . Contemporary staging and prognosis for primary tracheal Malignancies: a population-based analysis. Otolaryngol Head Neck Surg. (2004) 131:639–42. doi: 10.1016/j.otohns.2004.05.018. PMID: 15523440

[9] Wood DE . Management of Malignant tracheobronchial obstruction. Surg Clin North Am. (2002) 82:621–42. doi: 10.1016/s0039-6109(02)00025-7. PMID: 12371589

[10] Gaissert HA Grillo HC Shadmehr MB Wright CD Gokhale M Wain JC . Long-term survival after resection of primary adenoid cystic and squamous cell carcinoma of the trachea and carina. Ann Thorac Surg. (2004) 78:1889–96. doi: 10.1016/j.athoracsur.2004.05.064. PMID: 15560996

[11] Honings J van Dijck JA Verhagen AF van der Heijden HF Marres HA . Incidence and treatment of tracheal cancer: a nationwide study in the Netherlands. Ann Surg Oncol. (2007) 14:968–76. doi: 10.1245/s10434-006-9229-z. PMID: 17139460

[12] Ning Y He W Bian D Xie D Jiang G . Tracheo-bronchial adenoid cystic carcinoma: a retrospective study. Asia Pac J Clin Oncol. (2019) 15:244–9. doi: 10.1111/ajco.13162. PMID: 31111681

[13] Wen J Liu D Xu X Li S Chen X Zhou Q . Nomograms for predicting survival outcomes in patients with primary tracheal tumors: a large population-based analysis. Cancer Manag Res. (2018) 10:6843–56. doi: 10.2147/cmar.s186546. PMID: 30588090 PMC6294060

[14] Allen AM Rabin MS Reilly JJ Mentzer SJ . Unresectable adenoid cystic carcinoma of the trachea treated with chemoradiation. J Clin Oncol. (2007) 25:5521–3. doi: 10.1200/jco.2007.13.7273. PMID: 18048830

[15] Kniep HC Madesta F Schneider T Hanning U Schonfeld MH Schon G . Radiomics of brain MRI: utility in prediction of metastatic tumor type. Radiology. (2019) 290:479–87. doi: 10.1148/radiol.2018180946. PMID: 30526358

[16] He J Shen J Huang J Liu Q Wang H Li Y . Prognosis of primary tracheal tumor: a population-based analysis. J Surg Oncol. (2017) 115:1004–10. doi: 10.1002/jso.24611. PMID: 28407313

[17] Mallick S Benson R Giridhar P Rajan Singh A Rath GK . Demography, patterns of care and survival outcomes in patients with Malignant tumors of trachea: a systematic review and individual patient data analysis of 733 patients. Lung Cancer. (2019) 132:87–93. doi: 10.1016/j.lungcan.2019.04.017. PMID: 31097099

[18] Yang CJ Shah SA Ramakrishnan D Berry MF Detterbeck FC Boffa DJ . Impact of positive margins and radiation after tracheal adenoid cystic carcinoma resection on survival. Ann Thorac Surg. (2020) 109:1026–32. doi: 10.1016/j.athoracsur.2019.08.094. PMID: 31589850

[19] Shao D Gao Q Cheng Y Du DY Wang SY Wang SX . The prognostic value of (18)F-fluorodeoxyglucose PET/CT in the initial assessment of primary tracheal Malignant tumor: a retrospective study. Korean J Radiol. (2021) 22:425–34. doi: 10.3348/kjr.2020.0211. PMID: 33236543 PMC7909858

[20] Regnard JF Fourquier P Levasseur P . Results and prognostic factors in resections of primary tracheal tumors: a multicenter retrospective study. The French Society of Cardiovascular Surgery. J Thorac Cardiovasc Surg. (1996) 111:808–13. doi: 10.1201/9781315378596-9 8614141

[21] Maziak DE Todd TR Keshavjee SH Winton TL Van Nostrand P Pearson FG . Adenoid cystic carcinoma of the airway: thirty-two-year experience. J Thorac Cardiovasc Surg. (1996) 112:1522–31. doi: 10.1016/s0022-5223(96)70011-9. PMID: 8975844

[22] Luick ML Hansen EK Greenberg MS Cummings BJ Gospodarowicz MK Warde P . Primary tracheal non-Hodgkin's lymphoma. J Clin Oncol. (2011) 29:e193-195. doi: 10.1200/jco.2010.32.0309. PMID: 21172878

[23] Agrawal S Jackson C Celie KB Bumpous JM Padhya TA Shonka DC . Survival trends in patients with tracheal carcinoma from 1973 to 2011. Am J Otolaryngol. (2017) 38:673–7. doi: 10.1016/j.amjoto.2017.08.005. PMID: 28927948

[24] Xie L Fan M Sheets NC Chen RC Jiang GL Marks LB . The use of radiation therapy appears to improve outcome in patients with Malignant primary tracheal tumors: a SEER-based analysis. Int J Radiat Oncol Biol Phys. (2012) 84:464–70. doi: 10.1016/j.ijrobp.2011.12.011. PMID: 22365629

[25] Webb BD Walsh GL Roberts DB Sturgis EM . Primary tracheal Malignant neoplasms: the University of Texas MD Anderson Cancer Center experience. J Am Coll Surg. (2006) 202:237–46. doi: 10.1016/j.jamcollsurg.2005.09.016. PMID: 16427548

[26] Yusuf M Gaskins J Trawick E Reddy CA Videtic GMM Adelstein DJ . Effects of adjuvant radiation therapy on survival for patients with resected primary tracheal carcinoma: an analysis of the National Cancer Database. Jpn J Clin Oncol. (2019) 49:628–38. doi: 10.1016/j.ijrobp.2018.07.1805. PMID: 30977818

