# Supplementary material for: Development and validation of a CT-based radiomics nomogram for predicting overall survival in primary tracheal malignancy
Source: Front Oncol. 2026 Apr 15;16:1609920. doi: 10.3389/fonc.2026.1609920 (PMC13124618; doi:10.3389/fonc.2026.1609920)
Supplement: Supplementary Table 1 — Bootstrap validation results of the radiomics nomogram. [file Table1.docx]

Table S1. Bootstrap validation results of the radiomics nomogram.

| Metric | Original estimate | Bootstrap estimate | 95% CI |
| --- | --- | --- | --- |
| C-index | 0.79 | 0.76 | 0.70-0.82 |
| Hazard ratio (high-risk vs low-risk) | 3.12 | 2.95 | 1.80-4.50 |
| Log-rank p-value | < 0.001 | < 0.001 | — |
