# Supplementary figures and images for: Development and validation of a CT-based radiomics nomogram for predicting overall survival in primary tracheal malignancy
Source: Front Oncol. 2026 Apr 15;16:1609920. doi: 10.3389/fonc.2026.1609920 (PMC13124618; doi:10.3389/fonc.2026.1609920)

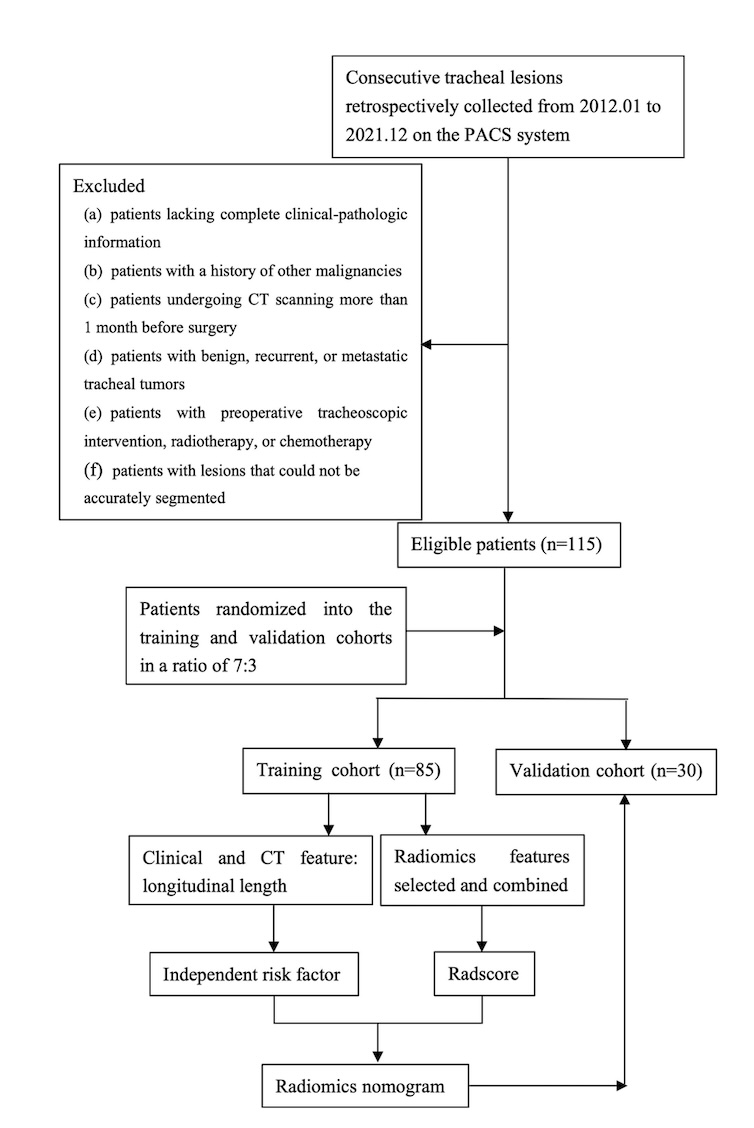

Supplement: Supplementary Figure 1 — Over-view workflow of this study. [file Image1.jpeg]

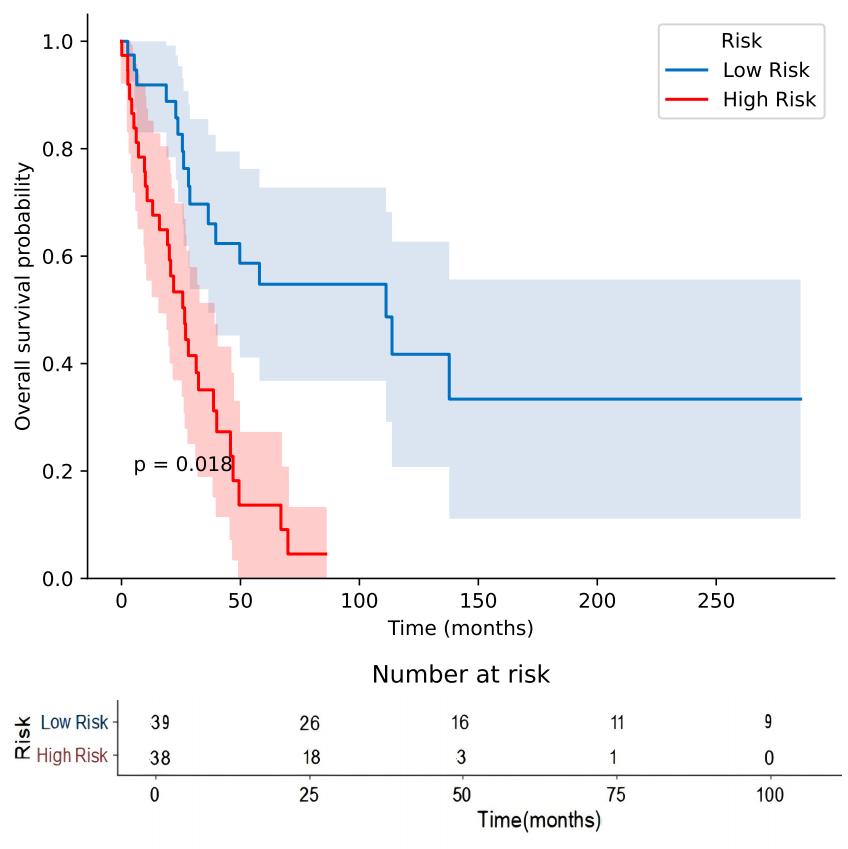

Supplement: Supplementary Figure 2 — Kaplan–Meier survival curves, with red and blue representing the high- and low-risk groups, respectively. The log-rank test showed a significant difference in overall survival between patients in the high- and low-risk groups within the adenoid cystic carcinoma (ACC) cohort (p = 0.018). [file Image2.jpeg]
